# Exploiting Vitamin D Receptor and Its Ligands to Target Squamous Cell Carcinomas of the Head and Neck

**DOI:** 10.3390/ijms24054675

**Published:** 2023-02-28

**Authors:** Laura Koll, Désirée Gül, Manal I. Elnouaem, Hanaa Raslan, Omneya R. Ramadan, Shirley K. Knauer, Sebastian Strieth, Jan Hagemann, Roland H. Stauber, Aya Khamis

**Affiliations:** 1 Department of Otorhinolaryngology Head and Neck Surgery, Molecular and Cellular Oncology, University Medical Center, 55131 Mainz, Germany; 2 Oral Pathology Department, Faculty of Dentistry, Alexandria University, Alexandria 5372066, Egypt; 3 Centre for Medical Biotechnology (ZMB/CENIDE), Institute for Molecular Biology, University Duisburg-Essen, Universitätsstraße, 45117 Essen, Germany; 4 Department of Otorhinolaryngology, University Medical Center Bonn, 53127 Bonn, Germany

**Keywords:** gender-specific effects, nuclear receptors, calcitriol, 3D tumor spheroids

## Abstract

Vitamin D (VitD) and its receptor (VDR) have been intensively investigated in many cancers. As knowledge for head and neck cancer (HNC) is limited, we investigated the (pre)clinical and therapeutic relevance of the VDR/VitD-axis. We found that VDR was differentially expressed in HNC tumors, correlating to the patients’ clinical parameters. Poorly differentiated tumors showed high VDR and Ki67 expression, whereas the VDR and Ki67 levels decreased from moderate to well-differentiated tumors. The VitD serum levels were lowest in patients with poorly differentiated cancers (4.1 ± 0.5 ng/mL), increasing from moderate (7.3 ± 4.3 ng/mL) to well-differentiated (13.2 ± 3.4 ng/mL) tumors. Notably, females showed higher VitD insufficiency compared to males, correlating with poor differentiation of the tumor. To mechanistically uncover VDR/VitD’s pathophysiological relevance, we demonstrated that VitD induced VDR nuclear-translocation (VitD < 100 nM) in HNC cells. RNA sequencing and heat map analysis showed that various nuclear receptors were differentially expressed in cisplatin-resistant versus sensitive HNC cells including VDR and the VDR interaction partner retinoic acid receptor (RXR). However, RXR expression was not significantly correlated with the clinical parameters, and cotreatment with its ligand, retinoic acid, did not enhance the killing by cisplatin. Moreover, the Chou–Talalay algorithm uncovered that VitD/cisplatin combinations synergistically killed tumor cells (VitD < 100 nM) and also inhibited the PI3K/Akt/mTOR pathway. Importantly, these findings were confirmed in 3D-tumor-spheroid models mimicking the patients’ tumor microarchitecture. Here, VitD already affected the 3D-tumor-spheroid formation, which was not seen in the 2D-cultures. We conclude that novel VDR/VitD-targeted drug combinations and nuclear receptors should also be intensely explored for HNC. Gender-specific VDR/VitD-effects may be correlated to socioeconomic differences and need to be considered during VitD (supplementation)-therapies.

## 1. Introduction

In the last three decades, there have been tremendous attempts to undercover the role of vitamin D (VitD) in the prevention, prognosis, and treatment of cancer. Unfortunately, the results have been contradictory, and until now, no general recommendations or standard treatment options considering VitD for cancer patients exist [1,2]. However, the majority of the observational studies supported a benefit of higher vitamin D intake concerning the reduction in cancer incidence (e.g., colon and breast cancer) [3,4]. Other studies showed a correlation between high serum VitD levels and lower cancer risk [5,6]. Nevertheless, for many entities, the clinical relevance of VitD as well as its molecular mechanisms of action requires further investigation.

Head and neck squamous cell carcinomas (HNC) are among the top ten most common cancers worldwide, frequently exhibiting limited treatment response [7,8,9,10]. Here, reasons for unsatisfactory treatment success and the long-term survival of HNC patients can be found in the development of resistance toward established treatments as well as the lack of novel therapeutic targets. A promising approach to potentially increase the success of established treatment options, which are surgery, chemo-, radiotherapy as well as targeted (Cetuximab) therapy, is the use of combinational treatments. Here, the use of functional foods such as VitD offers an alternative, cost-effective cancer care regimen harboring the potential to improve treatment success. Functional foods and food components affect the body, reaching beyond a basic nutritional effect. Among the group of functional foods, VitD is one of the most important members for which anti-tumoral effects have already been suggested [11,12]. Interestingly, little is known about the relevance of VitD for HNC patients in general and the potential clinical benefit of combinational VitD therapies in particular. Previous studies and meta-analyses have already demonstrated the need to determine and evaluate the VitD influence on cancer pathogenesis and patient prognosis [13,14].

VitD, which is rather a (steroid) hormone than a vitamin, has a variety of functions in health and disease [15]. Approximately 90% of the VitD requirement is produced in the skin in response to ultraviolet-B (UV-B) light from sun exposure. The biologically active form of VitD, 1α,25-dihydroxyVitD3 (1,25(OH)2D3), also called calcitriol, is produced by enzymatic conversions of its precursor calcidiol (25-hydroxy VitD, 25(OH)D3) via hydroxylation in the liver and kidneys [16]. 25-Hydroxy VitD is the most single reliable marker of VitD concentration in the body due to its relatively long half-life time (three weeks) compared to the active form of 1,25-OH2D (approximately 4 h) [15,16,17]. It is also an indication of the availability of the substrate for tissue production and the auto/paracrine action of 1,25-OH2D. On the other hand, although 1,25-OH2D is the active form, it is regulated by several enzymatic and physiological inputs [16]. The fact that 1,25-OH2D concentrations in the blood may not decrease or decrease at a late stage even in presence of VitD deficiency make 25-(OH)D3 a better marker for the assessment of VitD supply [18].

Notably, VitD deficiency is widespread and can cause various diseases such as rickets in children and osteoporosis [19]. Therefore, VitD food fortification is practiced in some countries. Moreover, VitD deficiency has been correlated to multiple systemic and physiological conditions such as insulin resistance and diabetes, autoimmune disease, cardiovascular disease, and all-cause mortality [16,20]. Importantly, VitD deficiency seems to be (in)directly correlated with the occurrence of cancer [21,22]. Furthermore, previous studies have suggested that sufficient levels of VitD can reduce the risk of many cancer types such as colon and breast cancer [3,4]. Meta-analyses show that patients with serum levels of the VitD pre-cursor calcidiol (25-hydroxyVitD) ranging from 20–40 ng/mL show a significantly reduced risk of about 35% for breast and colorectal cancer [23,24]. Furthermore, there is evidence for some entities such as breast, colorectal, lung, bladder cancer, and prostate cancer that higher serum levels of calcidiol at the time of diagnosis are correlated with improved survival rates [25,26,27,28,29].

VitD executes its biological functions via binding to the VitD receptor (VDR), a member of the nuclear receptor family [30,31]. Nuclear receptors (NRs) are key regulators of health and disease including cancer, and thus represent important targets for anti-cancer therapies [32,33,34]. NRs are transcription factors involved in a wide range and extremely complex spectrum of physiological and pathophysiological processes, hence they are interesting therapeutic targets [32,35]. However, despite intensive research, the detailed mechanisms of homo-/heterodimerized nuclear receptors including VDR are still not resolved (for more details, see also following reviews [3,4,32,34]. Upon the binding of its ligand VitD and nuclear translocation, VDR is able to form heterodimers with the retinoid X receptor (RXR). By binding to specific VDR-responsive elements on the DNA, the nuclear receptors are able to activate various transcriptional programs [3,4,30,31,32,34,36]. Importantly, for VDR, anti-tumoral effects have been already suggested [3,4,32,34,37,38].

Hence in this study, we investigated the (pre)clinical and potential therapeutic relevance of the VDR/VitD-axis to assess the association between the VitD level and VDR expression for HNC. Aside from analyzing the HNC dataset of The Cancer Genome Atlas (TCGA), a case-control study was analyzed. To mechanistically uncover VDR/VitD’s pathophysiological relevance, we further combined the evaluation of clinical data with comprehensive dry and wet lab systematic studies of innovative HNC cell models. Besides the use of the 2D tumor cell model, there is increasing evidence that advanced 3D tumor spheroids react differently compared to conventional 2D cultures when exposed to drugs, radiation, or signaling ligands [39,40,41,42]. Hence, we established a 3D cell culture model aiming to approach the tumor situation in vivo. In comparison to the 2D culture systems, 3D spheroids exhibit a number of advantages, for example, they mimic a more realistic 3D architecture of a tumor including the supply of nutrients, oxygen, and anti-cancer drug. Another advantage is the development of polarity in the spheroid culture due to neighboring cell-to-cell contacts [39,40]. Collectively, cells in 3D tumor spheroids seem to preserve key morphological and signaling patterns closely associated with tumor development and drug resistance in animal models and patients [39,40,41,42].

## 2. Results

### 2.1. VDR Expression and VitD Levels Correlate with HNC Patients’ Clinical Parameters

**Figure 1:**
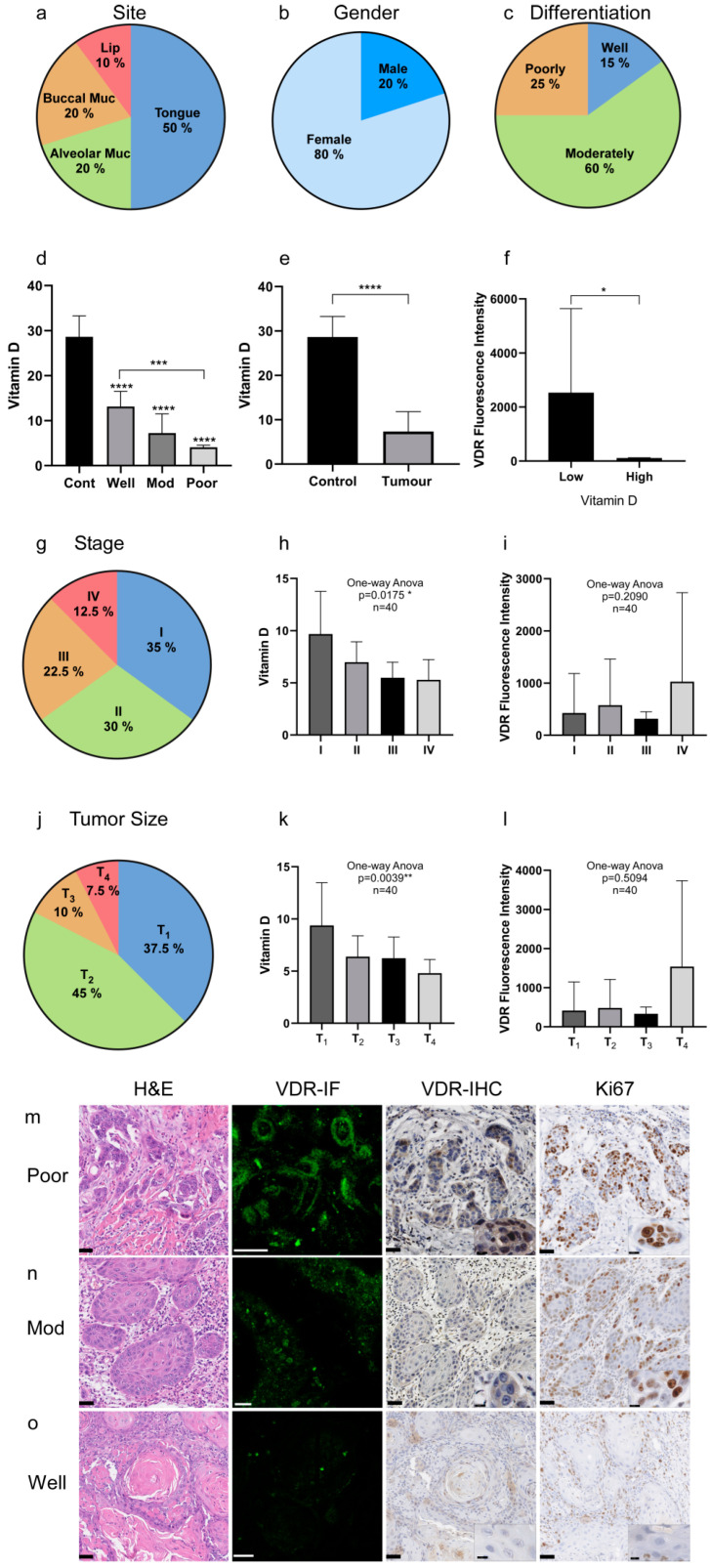
VDR expression and VitD levels correlate with the HNC patients’ clinical parameters. (**a**–**c**) Clinical characteristics of the HNC patient cohort (n = 40): (**a**) tumor site, (**b**) gender, (**c**) tumor differentiation. (**a**) The most common tumor type in this cohort occurred in the tongue followed by alveolar and buccal mucosa, and the lips. (**b**) The male–female ratio was 1:4. (**c**) Most of the analyzed tumors were classified as moderately differentiated. (**d**–**f**) Quantification of the VitD serum levels and corresponding VDR expression. (**d**,**e**) VitD (25-hydroxyVitD3) serum levels were determined in all individuals of the cohort, revealing significantly lower VitD levels in the tumor patients compared to the healthy controls. Here, patients with poorly differentiated tumors showed the lowest VitD levels. (**f**) VDR expression was inversely correlated with the VitD serum levels. (**g**–**i**) Staging of the HNSCC cases according to UICC (8th edition). (**g**) The most common stage was S-I with 35%, followed by S-II (30%), and S-III and S-IV with 22.5% and 12.5%, respectively. (**h**,**i**) Quantification and correlation of the VitD serum levels (**h**) and corresponding VDR expression (**i**). (**j**–**l**) Tumor size classification of HNSCC cases according to UICC (8th edition). (**j**) The most common subtype was T_1_ followed by T_2_ with 45% and 37.5%, respectively. Only a few cases were classified as T_3_ (10%) and T_4_ (7.5%). (**k**,**l**) Quantification and correlation of the VitD serum levels (**k**) and corresponding VDR expression (**l**). (**m**–**o**) High VDR expression occurred in poorly differentiated, highly proliferative tumor tissues. Expression of VDR and Ki67 was determined by immunofluorescence (IF) and immunohistochemical (IHC) staining of the tumor biopsies classified as poorly (**m**), moderately (**n**), and well-differentiated (**o**). IF staining of VDR (green) was visualized by confocal laser scanning microscopy, and the intensity of fluorescence (mean area percent, MA%) was measured using ImageJ (shown in (**f**)). Cells at higher magnification are included in the IHC image overviews. Representative examples are shown. Tissues were stained with H&E and specific Abs as indicated. Scale bars, 50 µm/12.5 µm (magnifications). Statistical significance is represented in figures as follows: * *p* < 0.05, ** *p* < 0.01, *** *p* < 0.001, and **** *p* < 0.0001. A *p* value that was less than 0.05 was considered statistically significant.

As knowledge of the VDR/VitD-axis for HNC is limited, we first investigated the VDR expression and VitD serum levels in a cohort of newly diagnosed HNC patients (n = 40) compared to the healthy individuals (n = 40) (details see Table 1, Supplementary Tables S3 and S4). The most common site of occurrence was the tongue (60%) and the least was the lip (Figure 1a). Notably, regarding gender, there was a significant difference in the male-to-female ratio (Figure 1b, Supplementary Tables S3 and S5) which is often observed in the Middle East and North Africa (MENA) region [43,44,45,46]. Histopathologically, the most common differentiation subtype was moderately differentiated HNC (60%, n = 24; Figure 1c, Supplementary Table S5).

In order to correlate the VitD serum levels with VDR expression in the tumor tissues, peripheral blood samples were taken from the patients before or during surgery. Total serum VitD (25-hydroxyVitD3) was quantified by using fully validated, modified high-performance liquid chromatography (HPLC) [47]. The VitD serum levels were lowest in patients with poorly differentiated cancers (4.1 ± 0.5 ng/mL), increasing from moderate (7.3 ± 4.3 ng/mL) to well-differentiated (13.2 ± 3.4 ng/mL) (Figure 1d, Table 2). The mean serum VitD level was 7.4 ± 4.5 ng/mL in cancer patients in comparison to 28.7 ± 4.6 ng/mL in healthy individuals (Figure 1e, Table 2). Notably, females showed higher VitD insufficiency compared to males, correlating with poor tumor differentiation (Table 1).

Additionally, the VDR protein expression was analyzed by immunofluorescence and immunohistochemical staining in tumor biopsies classified as poorly, moderately, and well-differentiated (Figure 1g–i). Here, a significant inversely proportional correlation between the VitD levels and VDR expression was found (Figure 1f). As shown in Figure 1g–i, all studied cases showed immunofluorescence reactivity to the VDR antibody with varying intensities. Moreover, we found that the VDR levels correlated with the patients’ clinical and pathobiological tumor parameters. Particularly, poorly differentiated tumors showed high VDR and Ki67 expression (Figure 1g), whereas VDR and Ki67 levels decreased from moderate to well-differentiated tumors (Figure 1h,i).

### 2.2. Clinical Relevance of VitD Receptor (VDR) and Retinoid X Receptor Alpha (RXRα) Expression in HNC Patients

To independently confirm the relevance of VDR expression in the HNC patients, we bioinformatically analyzed the PANCAN dataset acquired from The Cancer Genome Atlas (TCGA), encompassing more than 12,000 samples of cancer patients of various entities and clinical backgrounds. Moreover, upon binding of its ligand VitD and nuclear translocation, VDR is also able to form heterodimers with the retinoid X receptor (RXR), thereby activating various cancer-relevant transcriptional programs (Supplementary Figure S1) [23,25,32,34,48,49,50]. As VDR/RXR expression has not been studied for HNC, we also studied the expression of RXR in the datasets.

We found VDR overexpressed in the primary tumors. Comparing the different entities, the highest expression of VDR was found in rectal and colon adenocarcinoma and kidney cancer, directly followed by HNC (Supplementary Figure S2), supporting our conclusions obtained from the analyses of our cohort (see also Figure 1).

**Figure 2:**
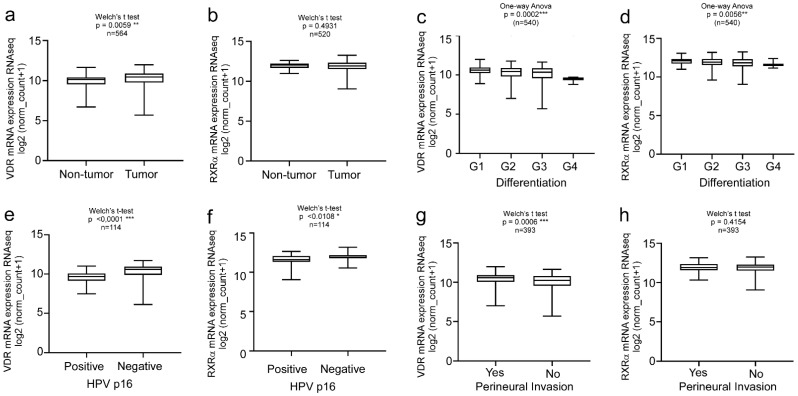
Clinical relevance of VDR vs. RXRα levels in HNC patients. Bioinformatics analysis of the TCGA HNC cohort (n = 604). Overexpression of VDR, but not RXRα was found in the primary tumors versus normal tissue (**a**,**b**) and correlates with (**c**) but to a lesser extent in RXRα. VDR expression correlates with tumor differentiation (**c**), negative HPV status (**e**), and perineural invasion (**g**). For all of the studies’ clinical parameters, RXRα showed less or no significant correlations compared to VDR (**d**,**f**,**h**). Significance *p* values and sample size (n) are indicated. Statistical significance is represented in figures as follows: * *p* < 0.05, ** *p* < 0.01 and *** *p* < 0.001. A *p* value that was less than 0.05 was considered statistically significant.

Thus, in the second step, we focused on the analysis of the TCGA HNC cohort (n = 604) showing upregulation of VDR in tumor versus non-tumor tissues (Figure 2a, n = 564, *p* = 0.0059 **). Interestingly, RXRα expression showed no correlation with the disease markers (Figure 2b, n = 520 *p* = 0.4931). VDR expression highly correlated with the histological differentiation of the tumor, in contrast to the RXRα levels (Figure 2c,d, n = 540, *p* = 0.0002 ***/*p* = 0.0056 **). Since the HPV status affects the therapy outcome and prognosis of HNC patients, we analyzed HPV-negative versus HPV-positive patients. VDR expression was significantly increased in HPV-negative HNC patients (Figure 2e, n = 114, *p* < 0.0001 ****). Again, changes in the RXRα levels were less significant (Figure 2f; n = 114; *p* < 0.0108). Moreover, high VDR expression correlated with perineural invasion (Figure 2g, n = 393, *p* = 0.0006 ***) in contrast to the RXRα levels (Figure 2h, n = 393, *p* = 0.4154), underlining again the relevance of VDR but not of RXRα as a biomarker and/or therapeutic target for HNC.

### 2.3. Nuclear Receptor Profiling and Translocation Kinetics in HNC Cells

**Figure 3:**
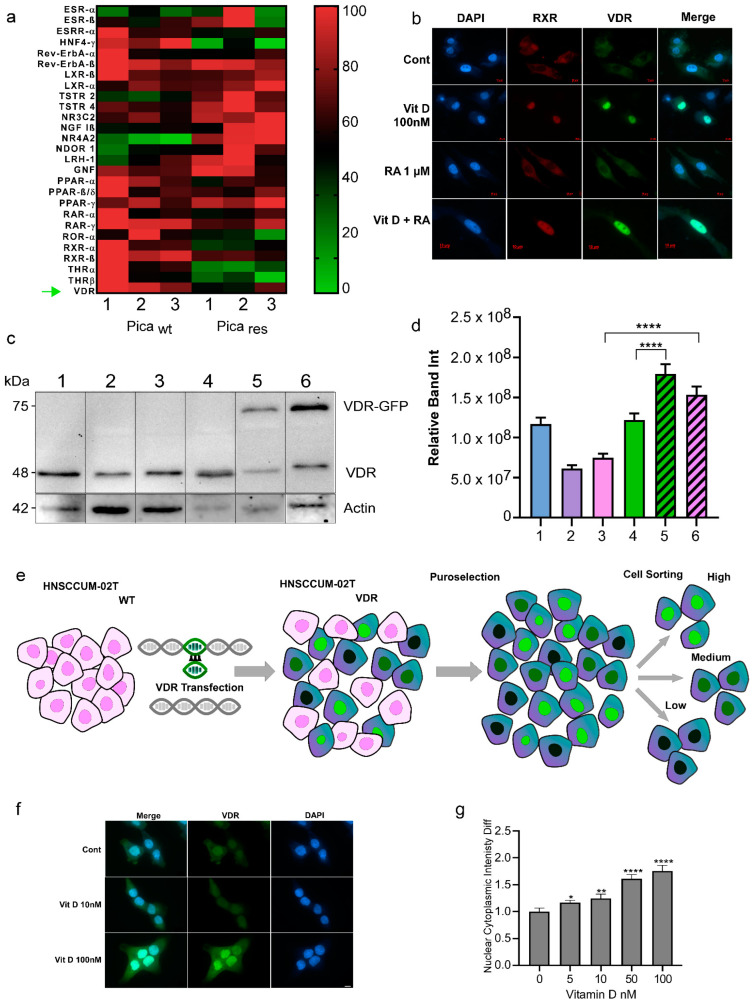
Nuclear receptor profiling in the HNC cell models. (**a**) RNA-Seq transcriptomics and heat map analysis illustrated that the expression of nuclear receptors is altered in therapy-resistant (res) versus sensitive (WT) Pica cells. A set of nuclear receptors were analyzed: Estrogen receptor-ß (ESR-ß), estrogen-related receptor-a (ESRR-α), hepatocyte nuclear factor-4-α (HNF4-α), rev-ErbAα, rev-ErbAß, liver X receptor-ß (LXR-ß), liver X receptor-α (LXR-α), testicular receptor 2 (TSTR 2), testicular receptor 4 (TSTR 4), mineralocorticoid receptor (NR3C2), nerve growth factor Iß (NGF Iß), Nuclear Receptor Subfamily 4 Group A Member 2 (NR4A2), neuron-derived orphan receptor 1 (NDOR 1), liver receptor homolog-1 (LRH-1), germ cell nuclear factor (GNF), peroxisome proliferator-activated receptor-α (PPAR-α), peroxisome proliferator-activated receptor-ß/δ (PPAR-ß/δ), retinoic acid receptor-α (RAR-α), retinoic acid receptor-γ (RAR-γ), RAR-related orphan receptor-α (ROR-α), retinoid X receptor-α (RXR-α), retinoid X receptor-ß (RXR-ß), thyroid hormone receptor-α (THR-α), and VitD receptor (VDR) ‘’green arrow’’. Heat map visualizes the expression levels of differentially expressed genes in WT vs. resistant cells (green: downregulated, red: upregulated). (**b**) Fluorescence microscopy to visualize VDR or RXR expression and activation/translocation in HNC cells. Cells were treated with 100 nM VitD, 1 µM retinoic acid (RA), or a combination of both (VitD + RA), for 30 min and fixed. Cells were stained with specific fluorescent VDR Ab (green), RXR Ab (red), and nuclei marked with Hoechst (blue). Scale bar, 10 µm. (**c**,**d**) Immunoblot quantification of endogenous VDR expression in the wt HNC cell lines (1-SCC-4, 2-HNCUM-01T, 3-HNCUM-02T, and 4-FaDu) as well as the VDR-GFP transfected, overexpressing cell lines (5-FaDu VDR, and 6-HNCUM-02T-VDR). Actin served as a loading control. (**d**) Relative protein quantification of the Western blot. (**e**) Generation of VDR-overexpressing HNC cell models. HNCUM-02T cells were transfected with VDR C-terminally fused to GFP (green). Positive clones were selected using puromycin and sorted by FACS into high, medium, and low subpopulations. Low-expressing subpopulations were used for the experiments. (**f**) Fluorescence microscopy visualized VitD-induced expression and nuclear translocation of VDR in HNC (HNCUM-02T) cells. Scale bar, 10 µm. Cells were treated with 10 or 100 nM VitD for 30 min, fixed and nuclei were stained with Hoechst (blue). (**g**) VDR translocation was automatically quantified by high-throughput microscopy and normalized to the untreated controls. Statistical analysis of the nucleus-to-cytoplasm ratio (N/C) revealed a significantly increased N/C ratio in the presence of VitD compared to the untreated controls, indicating a cytoplasm-to-nuclear translocation. Statistical significance is represented in figures as follows: * *p* < 0.05, ** *p* < 0.01, and **** *p* < 0.0001. A *p* value that was less than 0.05 was considered statistically significant.

It is accepted by the field that the superfamily of nuclear receptors are key regulators in many pathologies including cancer [32,33,34]. Thus, we used ‘omics’ approaches to profile nuclear receptor expression and the potential pathobiological relevance in HNC tumor cell models. As HNC treatment is often complicated by recurrence due to resistance to cisplatin-based treatments, we analyzed the chemoresistant HNC cells. The cisplatin-resistant cell line, Pica_res_, was established by selecting HNC Pica cells with sub-toxic concentrations of cisplatin (3–5 µM) for six months. Hence, Pica_wt_ and Pica_res_ allow for the comparison of cisplatin-sensitive and resistant HNC cells. Here, next-generation RNA sequencing transcriptomics was used to analyze the expression of various nuclear receptors (Figure 3a, Supplementary Table S6). As illustrated in the heat map analysis (Figure 3a; green: downregulated, red: upregulated), VDR and several other receptors such as Nuclear Receptor Subfamily 4 Group A Member 2 (NR4A2) or RXRα were differentially expressed in therapy-resistant (res) versus sensitive (wt) Pica cells. These data also suggest investigating the pathobiological relevance of other nuclear receptors for HNC in comprehensive follow-up studies.

When studying the impact of nuclear receptors, it is also the key to control if the respective receptor is expressed and indeed capable of cytoplasmic to nuclear trafficking upon ligand binding in the relevant cell model. Nuclear translocation is required to activate ligand-dependent transcriptional programs [3,4,30,31,32,34,36]. The activation of VDR by ligand binding typically involves VDR-RXRα dimerization and the initiation of downstream signaling (Supplementary Figure S1). The immunofluorescence staining of endogenous VDR and RXR receptors demonstrated that VitD triggered nuclear accumulation of the receptors, in contrast to retinoic acid (RA) treatment alone (Figure 3b). When referring to VitD, the active form calcitriol (1,25(OH)2D3) was used if not indicated otherwise. Hence, although both receptors are capable of cytoplasmic to nuclear trafficking, VitD and VDR seem more relevant in HNC cells. We also confirmed and quantified the VDR expression in different HNC cell lines (Figure 3c,d).

To further study the kinetics of VDR translocation in real-time, we established HNC cell lines stably expressing VDR fused to GFP. Therefore, the VDR reading frame was cloned from primary HNC tumor cells and stably expressed VDR-GFP in the HNCUM-02T or HNC FaDu cells (Figure 3e). An important question regarding VDR’s nuclear translocation is the determination of the most effective ligand dose and the time kinetics of the process. Using the high-content screening microscopy platform, Array Scan VTI, we automatically quantified VDR translocation. Here, cells were treated with different clinically relevant doses of VitD (0–100 nM) for 30 min (Figure 3f,g). Fluorescence microscopy showed dose-dependent VDR translocation into the nucleus by VitD, which was most effective at a VitD concentration of 100 nM. Importantly, RA alone did not trigger the nuclear translocation of VDR (Supplementary Figure S3).

### 2.4. VitD/VDR Targeting Synergistically Improves Cisplatin-Mediated Killing of HNC Tumor Cells

Chemoresistance is not only one of the main causes influencing cancer progression, but it is also strongly correlated to the cancer mortality rates. Hence, developing strategies for enhancing chemo sensitivity, potentially also by functional food supplementation with VitD, is expected to benefit patients. Indeed, such efforts have been made to correct VitD deficiency in cancer patients [11,12,51,52]. However, the success of VitD/VDR targeting therapies requires mechanistic knowledge and experimental investigation in vitro.

**Figure 4:**
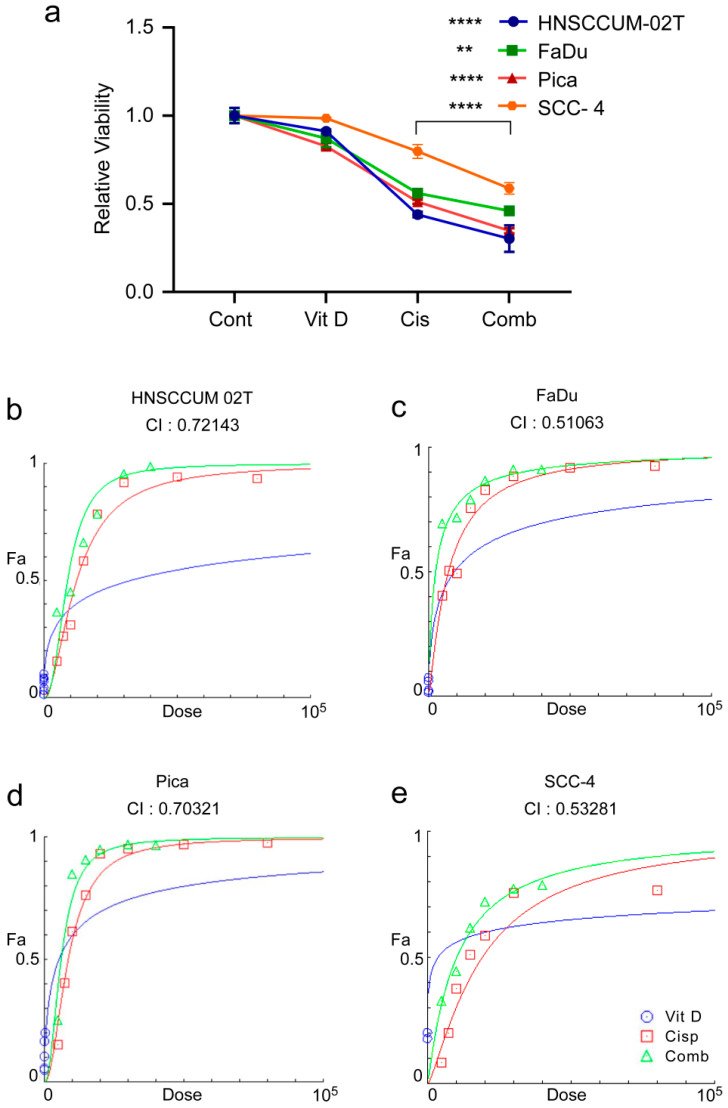
VitD/cisplatin combinations synergistically enhance the cisplatin-mediated killing of HNC tumor cells. (**a**) Cotreatment of VitD/cisplatin synergistically triggered the cell death of HNCUM-02T, FaDu, Pica, and SCC-4 cells compared to cisplatin alone. Cells were seeded in the presence of 100 nM VitD, and after 24 h, the cells were treated with 15–20 µM cisplatin with or without VitD (100 nM). The viability of the untreated control was set to 1. (**b**–**e**) Chou–Talalay dose–effect curves and calculation of the combination index (CI) demonstrate the synergistic effect of VitD/cisplatin combination treatments in HNCUM-02T, FaDu, Pica cells, and SCC-4, respectively. ** *p* < 0.01 and **** *p* < 0.0001.

To examine the effect of combination therapy on HNC, we thus measured the cell viability after the VitD/cisplatin treatments. To also mimic the pathophysiological conditions of high and low VitD serum levels, cells were seeded in the presence or absence of 100 nM VitD, which we found to trigger efficient VDR nuclear translocation, and thus biological activation (see Figure 3f,g). After 24 h of VitD pre-treatment, cells were additionally treated with physiological concentrations of VitD (100 nM), 15–20 µM cisplatin, or a combination (Figure 4). As expected, VitD alone did not affect cell viability. However, the combination treatments significantly enhanced tumor cell death compared to cisplatin alone in the three HNC cell lines tested (Figure 4a; Supplementary Figure S4). To objectively uncover a potential synergistic effect of VitD/cisplatin combinational treatments, we performed the Chou–Talalay method. The calculation of the combination index (CI) using the Chou–Talalay algorithm allowed us to uncover additive (CI = 1), synergistic (CI < 1), or antagonistic effects (CI > 1) of the drug combinations [53]. As shown in Figure 4b–d, all calculated indices were less than 1, revealing a synergistic effect on tumor cell killing for the VitD/cisplatin combinations in the tested HNCUM 02T, FaDu, and Pica cell lines.

### 2.5. Impact of VitD/VDR Targeting on HNC 3D Tumor Spheroids

Conventional 2D tumor cell models are well-established tools to assess various aspects of tumor pathobiology. However, there is increasing evidence that advanced 3D tumor spheroids react differently compared to conventional 2D cultures when exposed to drugs, radiation, or signaling ligands [39,40]. The architecture of spheroids leads to a gradient of nutrition and oxygen from the outer surface to the core, and drug delivery to parts of the 3D cell cluster also seems to differ. Additionally, cells in 3D tumor spheroids seem to preserve certain distinct signaling patterns that are closely associated with drug resistance in animal models and patients.

**Figure 5:**
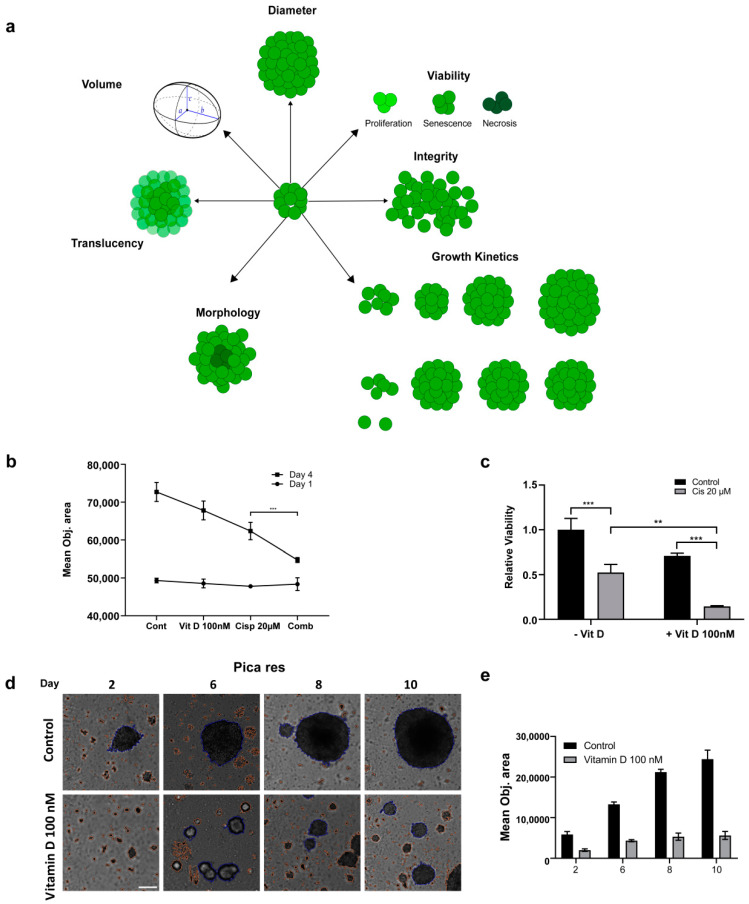
Assessing the impact of VitD/VDR targeting in HNC 3D tumor spheroids. (**a**) Illustration depicting different 3D spheroid properties that can be assessed using label-free, automated high-content microscopy. (**b**) The synergistic killing effect of VitD/cisplatin reduced the growth of the Pica_res_ 3D spheroids. The mean object sizes of the spheroids (n = 8) were automatically determined by HCS microscopy (Array Scan VTI) following VitD (100 nM), cisplatin (20 µM), or a combination of both for 72 h and normalized to the untreated control. (**c**) The synergistic killing effect of the VitD/cisplatin combinations was also reflected by the cell viability of the Pica_res_ 3D spheroids. Spheroids were treated as described in (**b**). **, *p* < 0.01, ***, *p* < 0.005 (**d**,**e**) VitD treatment affected 3D spheroid formation. Automated high-content microscopy was used to visualize (**d**) and quantify (**e**) the Pica_res_ tumor spheroid growth after VitD treatment. Spheroid formation was observed for 10 days with and without (control) the addition of 100 nM VitD. The mean object area of the Pica_res_ spheroids (n = 8) was automatically determined by HCS Array Scan VTI. Representative spheroids are shown. Scale bar 250 µm.

In order to closely approach the tumor situation in vivo, we next established HNC 3D tumor spheroids to investigate the effects of VitD/VDR targeting in an experimental setting, more closely mimicking the patients’ tumor microenvironments. Here, cells were cultured in ultra-low adhesion cell culture vials that promoted the formation of 3D spheroid-shaped tumor cell clusters. As summarized in Figure 5a, various pathobiological relevant properties of the established 3D spheroids were subsequently analyzed by fully automated high-content microscopy, allowing for an objective assessment of the tumor spheroids’ growth, morphology, and vitality.

First, we found that the synergistic killing effect of the VitD/cisplatin combinations observed in the 2D cultures was also relevant for the 3D spheroids (Figure 5b). Cotreatment significantly reduced the mean objective area and viability (Figure 5b,c). Interestingly, although VitD alone did not affect the vitality of the 2D cultures, it already affected the 3D spheroid formation and induced morphological and architectural changes. As shown in Figure 5b–e and Supplementary Figure S5, automated high-content microscopy revealed that spheroid formation and growth were significantly impaired, suggesting that the expression of epithelial surface markers may be reduced. Notably, the effect was more prominent for the cisplatin-resistant cell line Pica_res_ (Figure 5d,e, Supplementary Figure S5), although the molecular details are not known. In conclusion, these data uncover a novel effect of VitD and also demonstrate that 3D tumor spheroids are a valuable experimental tool to uncover aspects of tumor pathobiology potentially occluded in conventional 2D tumor cell models.

### 2.6. VitD Enhances the Chemotherapeutic Effect via mTOR-PI3K/Akt Downregulation in HNC

**Figure 6:**
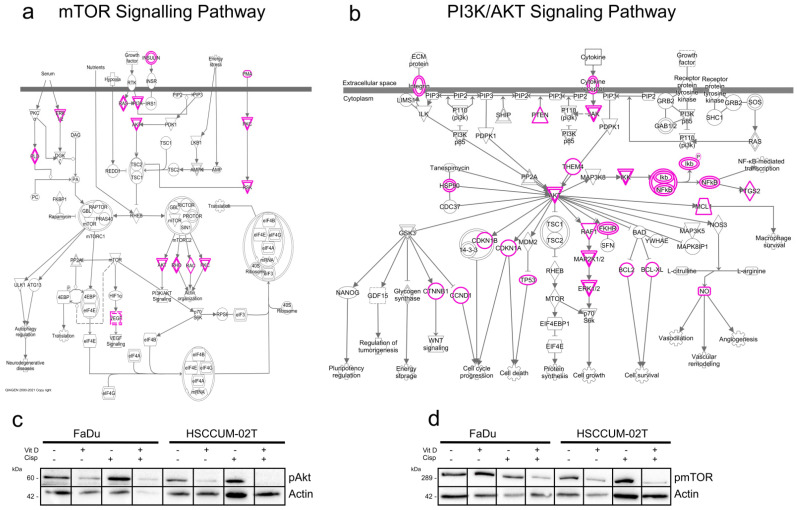
VitD enhances the chemotherapeutic effect of cisplatin via mTOR-PI3K/Akt downregulation. (**a**,**b**) Ingenuity pathway analysis summarizing the mTOR (**a**) and PI3K/Akt (**b**) signaling pathways in cancer. Proteins potentially affected by VitD/VDR activation are highlighted in pink. (**c**,**d**) Immunoblot analyses revealed a significant decrease in pmTOR (**c**) and pAkt (**d**) in the VitD/cisplatin co-treated FaDu and HNCUM-02T cells. Actin served as a loading control. Proteins were detected by specific Abs.

To further investigate how VitD or VitD/cisplatin combinations inhibit the proliferation and clonogenic survival of HNC cells, we examined the cancer-relevant signaling pathways. First, bioinformatics analyses employing the Ingenuity Pathway Analysis software (Version v01-04) revealed multiple molecular mechanisms involved in cancer pathogenesis and treatment resistance (Supplementary Figure S6). Subsequently, we focused on potential VDR-RXR activation pathways (Supplementary Figure S1) and further explored the literature [54,55,56,57]. As summarized in Figure 6a,b, VitD has been suggested to regulate several pathways including the cancer-relevant mTOR/PI3K-Akt pathways. Here, key regulatory proteins are (in)directly affected by VitD overlap such as the Akt kinase (Figure 6a,b). Under ‘healthy’ conditions, the mTOR/PI3K-Akt pathways are important players in development, cellular homeostasis, and health control.

However, in cancer, abnormally activated mTOR/PI3K-Akt signaling stimulates tumor cells to grow, metastasize, and become resistant to treatment [54,55,56,57,58]. Notably, when we examined the impact of VitD and VitD/cisplatin treatment combinations in HNC cell models, we found that expression of the active, phosphorylated forms of mTOR and Akt (i.e., of pmTOR and pAkt) was particularly decreased upon VitD/cisplatin cotreatment (Figure 6c,d). No significant reduction in pmTOR and pAkt was detected upon cisplatin treatment alone. These findings not only provide a potential molecular explanation for the enhanced cisplatin-killing effect on the cancer cells by VitD, but also suggest the further experimental exploitation of additional cotreatment combinations such as using mTOR and Akt inhibitors in combination with VitD.

## 3. Discussion

The VDR/VitD-axis has been intensively investigated for more than a decade for the prevention and/or treatment of many cancers. Such (pre)clinical studies range from VitD food supplementation and cancer-prevention trials to different combination therapies [3,4,32,34,37]. Indeed, various anti-tumoral effects have been suggested for this member of the nuclear receptor superfamily, and VitD deficiency is often observed in cancer patients [21,22,59,60]. However, the underlying mechanisms of the VitD/VDR-mediated effects are not understood in detail and sometimes conflicting reports underline that its role, especially in specific cancer types, remains to be dissected [3,4,34,37].

Our clinical and experimental data support a significant role of the VDR/VitD-axis in the prognosis and clinical outcome of HNC patients. First, by analyzing our cohort of n = 40 HNC patients compared to healthy controls (n = 40), we demonstrated that both the VitD serum levels and VDR expression correlate with clinical parameters such as histopathological tumor classification. Although we could not provide specific data on patient prognoses such as survival curves, in general, the HNC patients’ overall survival correlates with histopathological differentiation of the tumor (see Supplementary Figure S7). Our finding that patients with poorly differentiated tumors and thus poor prognosis exhibited the lowest VitD levels is in line with previous studies of other entities. For example, Yao et al. found that low serum 25OHD levels at diagnosis were associated with poorer survival and worse prognosis in breast cancer patients [61]. Additionally, there have been studies observing an inverse relationship between cancer mortality and serum VitD level [59,62], suggesting that VitD supplementation therapy was most effective in patients with VitD deficiency at diagnosis [62]. However, in contrast to other clinical studies, we here paid attention to recruiting an age-sex-matched control group of non-cancer patients, allowing us to draw conclusions about a potential (gender-specific) correlation between the serum VitD level and HNC. This study’s confinement of cases to 40 patients due to the complexity of the subject matter could be seen as a potential limitation. It also has to be mentioned that the study cohort includes tumors of different sites such as the tongue and lip, which can differ in their prognosis. Nevertheless, the cohort is suitable to represent the commonly observed distribution of subsites and histopathological differentiation.

Of note, our study cohort was recruited in Egypt, exhibiting socio-economical characteristics, which we feel worth discussing. First, the study cohort differed in its gender composition from typical Northern European and American study groups because it consisted mainly of women (male–female ratio 1:4). This is often observed in the Middle East and North Africa (MENA) region [43,44,45,46], which among other factors such as increased smoking [63,64] could be explained by differences in VitD supply. A normal VitD supply is defined as when the 25(OH)D serum concentrations ranged between 30 and 50 ng/mL, whereas levels <20 ng/mL were classified as VitD deficiency [65,66]. The mean level of serum VitD (25(OH)D) in the healthy population differs depending on the geographical residence, whereas mean VitD levels in adults in North America, Asia Pacific, and Europe range between 20.4 and 28.9 ng/mL, and thus could be classified as insufficient, but not yet deficient. Interestingly, in the Middle East and North Africa region (MENA) the mean VitD levels seem to be significantly lower with 13.6–15.2 ng/mL (applies to the same age group, does not take differences in sunshine duration into account) [67]. Different reasons may explain lower VitD levels in the MENA region such as increased air pollution, reducing the amount of UVB rays available for VitD production in the skin [17,68,69]. Another explanation could be a physiological de-toxification mechanism of VitD, which is activated after longtime sunlight exposure to prevent the toxic effects of very high VitD levels in the human body [15,16]. While the analyzed healthy patients of our study cohort lay above the statistic MENA value with a mean VitD concentration of 28.7 ng/mL, the HNC patients exhibited very low VitD levels (mean 7.4 ng/mL), classified as severe VitD deficiency (<12 ng/mL). Here, especially the female patients exhibited very low VitD levels (5.3 ng/mL), which is supported by other studies describing the female gender as a risk factor for hypovitaminosis [67]. Aside from the general reasons for VitD deficiency in the MENA region described above, additional circumstances such as veiling and/or reserved clothing style, lower socio-economic standard, and predominant indoor activity may contribute to VitD deficiency in women [67,70,71]. These factors come along with the lack of awareness about the importance of VitD to the human body [67]. Assuming a significant role of VitD in the pathogenesis of HNC, this could partly explain the increased incidence of HNC in females. Such a correlation has also been suggested for colorectal cancer. Here, the VitD levels were inversely proportional to the risk of cancer in women, but not statistically significant in men [24]. Furthermore, it has been proposed that VitD supplementation could be protective against breast cancer in menopausal women, underlining its effect on tumorigenesis [72]. Again, for the gender-specific conclusions also drawn from our study, the restricted sample size of n = 34 females should be considered, suggesting further larger studies focusing on the gender-specific relevance of VitD in HNC.

Since VitD executes its biological functions via nuclear receptor binding, we analyzed the clinical relevance as well as expression and ligand-dependent activation of VDR and its heterodimerization partner RXRα. Here, we showed that VDR, but not RXRα, was significantly overexpressed in the primary HNC patients, which also correlated with clinically relevant disease markers such as HPV status, perineural invasion, and histopathological differentiation. However, there are some conflicting studies about the clinical relevance of VDR overexpression for tumorigenesis [73,74,75]. For example, Choi et al. correlated the VDR overexpression with negative prognosis in thyroid cancer [73], supporting our data showing that VDR overexpression in poorly differentiated, highly proliferative tumor tissue. Other studies have correlated the high VDR expression with an improved prognosis of patients [74,75,76]. RXRα expression and its clinical relevance in HNC have also been controversially discussed. RXR agonists such as bexarotene can benefit HPV-negative HNC patients [77]. Bexarotene combination therapy was also effective in a preclinical trial [78]. For breast cancer, there are studies demonstrating a concurrent overexpression of VDR and RXRα [79], partially also describing a worse disease-free survival when RXRα is overexpressed [80,81,82]. Hence, RXR might be worth investigating in future experimental and clinical VitD/VDR studies in general.

Chemoresistance is a major cause of cancer progression and impacts the mortality of cancer patients, particularly for HNC [9,10,39,83]. Hence, developing strategies for enhancing chemosensitivity, potentially also by food supplementation with VitD, is needed and may benefit patients. Indeed, such efforts have been made to correct VitD deficiency in cancer patients [11,12,51,52]. Through our comprehensive in vitro studies applying established 2D as well as 3D spheroid HNC cell models, we could show that VitD treatment improves chemotherapeutic killing, especially of therapy-resistant HNC tumor cells, suggesting VitD supplementation during the primary (radio)chemotherapy of HNC patients. Of course, the serum VitD levels of respective patients should be carefully monitored during therapy, and other clinically relevant factors also have to be considered. Previous observational studies and clinical trials have partially reported improved survival of cancer patients after VitD supplementation, but the findings are not conclusive yet, and further studies combining clinical with wet lab investigation are needed [84].

Our nuclear receptor profiling by next-generation RNA sequencing transcriptomics provides the first data suggesting that other nuclear receptors may also be relevant for cisplatin-chemoresistance in HNC. Furthermore, VDR or RXRα investigated here, differentially expressed receptors such as Nuclear Receptor Subfamily 4 Group A Member 2 (NR4A2) seem to be relevant for various aspects of HNC pathobiology including HPV status and mTOR/Akt signaling, underlining the value of our datasets [85,86,87]. Due to the complexity of this area, we did not explore other nuclear receptors in this study, which might be considered as a potential limitation. Hence, we refer the reader to the literature regarding the specific receptor of interest. We conclude that the data provided here may stimulate the field to further explore the relevance of the nuclear receptor superfamily for therapy resistance in HNC.

In cancer, abnormally (de)activated signaling pathways such as mTOR/PI3K-Akt and NFκB signaling stimulate tumor cells to proliferate aggressively, metastasize, and become even more resilient to therapy [54,55,56,57,58]. Here, we found that the VitD/VDR-axis enhances the chemotherapeutic effect via mTOR-PI3K/Akt downregulation in HNC. The potential relevance of the Akt- and mTOR pathways in VitD/VDR signaling is supported by reports in other tumor types [56,88]. It has to be mentioned that VitD executes its biological functions by various cellular pathways, and thus is likely that additional proapoptotic pathways may contribute to cancer-associated VitD effects. Of note, bioinformatic modeling and predictions, as performed in our study, will aid in hypothesis building, but detailed investigations are needed to confirm the candidates’ relevance. Our findings not only suggest an additional molecular mechanism for the observed beneficial effects of VitD supplementation, but also suggest further exploitation of additional cotreatment combinations such as using mTOR and Akt inhibitors (e.g., ICSN3250, LY3023414, AZD8055, or rapamycin) [58,89]. However, these preliminary results give the first molecular evidence for further co-treatment options, and detailed analyses have to be performed in future (pre-)clinical studies.

Collectively, we can conclude that novel VDR/VitD-aided drug combinations should be intensely investigated in (pre)clinical studies. Here, gender-specific VDR/VitD-effects impacted by country-specific socioeconomic differences may need additional attention. Moreover, nuclear receptors should be further explored not only for breast or colon cancer, but also for HNC.

## 4. Materials and Methods

### 4.1. Chemicals and Reagents

Unless stated otherwise, chemicals were purchased from Sigma Aldrich/Merck (Darmstadt/Munich, Germany) or MSC (MSC UG and CoKG, Mainz, Germany). Cell culture media and reagents were sourced from Gibco/Thermo Fisher Scientific (Dreieich, Germany). Disposables were purchased from Greiner Bio-One (Frickenhausen, Germany). Ab used: α-VDR (sc-13133; Santa Cruz Biotechnology, Heidelberg, Germany), α-VDR (ab3508, Abcam, Erlangen, Germany), α-RXRα (5388, Cell Signaling, Leiden, The Netherlands), α-phospho-mTOR (5536, Cell Signaling, Leiden, The Netherlands), α-phospho-Akt (3787, Cell Signaling, Leiden, The Netherlands), and α-actin (A2066; Sigma Aldrich, Munich, Germany). Appropriate HRP-, Cy3-, or FITC-conjugated secondary antibodies (Sigma Aldrich, Munich, Germany; Santa Cruz Biotechnology, Heidelberg, Germany) were used (Supplementary Table S1). Reagents such as cisplatin were from Sigma (Sigma Aldrich, Munich, Germany) or MSC (MSC UG & CoKG, Mainz, Germany). 1α,25-Dihydroxy VitD_3_ (Calcitriol) was purchased from Sigma and Santa-Cruz (D1530, Sigma Aldrich, Munich, Germany and CAS 32222-06-3, Santa Cruz Biotechnology, Heidelberg, Germany). Ki67 (IR626, DAKO Agilent, Santa Clara, CA, USA), and DakoEnVision Flex (Linker) (DM824, DAKO Agilent, Santa Clara, CA, USA) were also used.

### 4.2. Study Population

The investigation was conducted following the ethical standards according to the Declaration of Helsinki of 1975 and according to the local, national, and international guidelines. Tissue samples were obtained from patients undergoing surgical resection of HNC at the Department of Oral and Maxillofacial Surgery at the Faculty of Dentistry of Alexandria University from December 2017 to November 2018. In that period, the cases were consecutively enrolled in the study. The study protocol was approved by the local ethics committee (#0008839) after obtaining the patient’s informed consent to participate in the study and was processed anonymously. Patients undergoing simultaneous chemo- or radio-treatment before or during the surgery were excluded from the study. All cases were diagnosed histopathologically as HNC and staged according to the TNM classification of malignant tumors recommended by the ‘Union International Contre le Cancer UICC (8th edition). All experiments were performed in accordance with the relevant laws and the Alexandria University Guidelines and approved by the institutional ethics committee at the Faculty of Dentistry, Alexandria University. In this study, tumor specimens and corresponding non-malignant tissue were analyzed, different tumor sizes (T1–T4), lymph node status (N0-2), and grading G1–G3. Upon resection, samples were immediately fixed in formaldehyde. Histological analyses were performed to ensure that each specimen contained >70% tumor tissue and <10% necrotic debris. Samples not meeting these criteria were rejected. Specimens were handled as usual (i.e., paraffin-embedded, sectioned, and H&E staining). The H&E stain was implemented by staining the specimens with Harris’ hematoxylin as described [83,90]. The interpretation was performed by oral pathologists at Alexandria University. Peripheral blood samples were taken from patients before or during surgery. The total serum 25-hydroxyVitD concentration (sum of D3 & D2 forms) is regarded as the best single marker of VitD status in the human body. Total serum VitD (25-hydroxyVitD3) was quantified by using fully validated, modified high-performance liquid chromatography (HPLC) [47].

### 4.3. Clinical Data Analysis

HNC tissue samples were included from The Cancer Genome Atlas (TCGA) Research Network (http://cancergenome.nih.gov/, accessed on 1 October 2022). The TCGA Research Network included patients following the guidelines of the Declaration of Helsinki of 1975 and all patients provided signed informed consent.

Publicly available gene expression and survival datasets were obtained from The Cancer Genome Atlas (TCGA) Research Network (http://cancergenome.nih.gov/, accessed on 1 October 2022), filtering for patients with HNCs (TCGA HNC). Of note, the expression values were not detectable for all genes of interest for every patient in the TCGA database. Here, VDR and RXR expression was found in n = 604 patients and analyzed as described [39]. Data were assessed via the USCS Xena server and patients were grouped according to the indicated phenotypic or clinical characteristics as described [37].

### 4.4. Cell Culture

Authenticated and characterized cell lines FaDu and SCC-4 were purchased from the *ATCC* repository, expanded, stocks prepared at early passages, and frozen stocks kept in liquid nitrogen. SCC-4 cells were established from a tongue squamous cell carcinoma. HNCUM-01T and -HNCUM-02T were established from tongue squamous cell carcinoma as described by Welkoborsky et al. [91]. The Pica cell line was established from laryngeal squamous cell carcinoma and maintained as described [39]. The FaDu cell line was established from a hypopharyngeal squamous cell carcinoma [92]. Thawed cells were routinely monitored by visual inspection and growth-curve analyses to keep track of the cell-doubling times, and were used for a maximum of 20 passages for all experiments. Depending on the passage number from purchase, cell line authentication was further performed at reasonable intervals by short tandem repeat (STR) profiling. We cultured the HNCUM-01T, HNCUM-02T, and SCC-4 cells in Dulbecco’s modified Eagle’s F-12 medium. Pica and FaDu cells were cultured in Dulbecco’s modified Eagle’s medium. We added 10% fetal bovine serum (FBS), and 1% penicillin-streptomycin to all medium types. Cells were cultured under a 5% CO_2_ atmosphere at 37 °C and subcultured every 3 days as described [39]. We checked the absence of mycoplasma regularly via the Venor GeM Advance Detection Kit (Minverva Biolabs, Berlin, Germany) according to the manufacturer’s instructions. The cell numbers were determined using Casy Cell Counter and Analyzer TT (OMNI Life Science GmbH & Co KG, Bremen, Germany). To treat the cells, Hy-clone fetal bovine serum (FBS) (Sigma Aldrich, Munich, Germany) was used instead of standard FBS to ensure the absence of VitD in the controls and the control VitD treatment doses in the treated samples.

### 4.5. Generation of Cisplatin Resistant Cell Model

We generated constantly selected cell lines by treatment with sub-toxic doses of cisplatin corresponding to IC90 (5 µM) and then constant treatment (3 µM). We used the resistant cell line for experiments 6 months after constant exposure to cisplatin and the re-establishment of relatively regular proliferation.

### 4.6. Cell Viability Assays

To probe cell viability, we seeded the cells in 96-well plates (5000 to 15,000 cells/well) depending on the cell line and the treatment duration and treated them with the indicated substances and concentrations (n = 3) starting 24 h after seeding. After 48/72 h treatment, we performed a commercially available assay CellTiter-Glo^®^ 2.0 (Promega, Walldorf, Germany) according to the manufacturer’s instructions and recorded the luminescent signals using a Tecan Spark^®^ (Tecan Group Ltd., Männedorf, Switzerland). Later, we normalized the signals to the untreated control samples.

In order to objectively determine the pharmacological effect of the proposed drug combination, we used the combination index equation described by Chou–Talalay [53]. In this algorithm, synergy is defined as combination index values < 1.0, antagonism as values > 1.0, and additivity as a value = 1.0.

### 4.7. Fluorescence Microscopy

Fluorescence images were acquired, analyzed, and quantified using an Axiovert 200 M fluorescence microscope (Zeiss, Oberkochen, Germany) or an automated high-content screening microscope Array Scan VTI (Thermo Fisher, Dreieich, Germany) as described [39,93,94]. We seeded cells in microscopic dishes (35 mm, MatTek, Ashland, MA, USA) or clear-bottom 96-well plates (Greiner, Kremsmünster Austria) and fixed them with 4% PFA (20 min, RT). For immunofluorescence staining, we additionally permeabilized the cells via incubation with Triton-X 100 (0.1%, 10 min, RT). Antibodies were diluted in 10% FBS/PBS and incubated with samples for 1 h at RT. We washed the cells (n = 3) in PBS and then incubated the samples with fluorophore-labeled antibodies for 1 h at RT. Finally, we stained the nuclei by adding Hoechst 33342 (50 ng/mL in PBS) for 30 min at RT. For automated high-content screening, regions of interest were created using the nucleus signal and each sample was acquired in triplicate, imaging at least 5000 events per sample according to [39].

### 4.8. RNA Sequencing and Visualization

RNA sequencing was then performed as described in [95] and the visualizations were achieved with the help of GraphPad Prism. Ingenuity Pathway Analysis (Qiagen, Hilden, Germany) was used to visualize the mTOR and PI3K-AKT signaling pathways.

### 4.9. Plasmids and Transfection

To construct a VDR expression plasmid, cDNA was isolated out of the HNC cancer cell lines, and the full open reading frame of human VDR cDNA was cloned into the pcDNA3.1 mammalian expression vector (Invitrogen, Karlsruhe, Germany) with the C-terminal GFP-tag (for primer sequences, please see Supplementary Table S2). Colony PCR was performed to check for positive clones [93,94,96].

For cellular transfection, plasmid DNA and Lipofectamine 3000 (Fisher Scientific, Schwerte, Germany) were mixed according to the manufacturer’s instructions and added to the cells, which were cultured in Opti-MEM medium as described [97].To mark VDR-expressing cells, plasmid pC3 coding for GFP expression was co-transfected. To exclude artifacts, a control transfection of empty plasmid pC-DNA3 and the GFP-coding plasmid was conducted in parallel. The medium was changed 5 h post-transfection to a normal cell culture medium. We confirmed the VDR overexpression of cell lines via Western blot analysis, and positively transfected cells were selected by the addition of puromycin (1 µg/mL; Sigma Aldrich, Munich, Germany). To establish a uniform expression of the VDR transfected cells, the cells were sorted into low, medium, and high fluorescence using FACS as previously described [96].

### 4.10. Protein Extraction, Immunoblot Analysis

Whole-cell lysates were prepared using low salt lysis RIPA buffer (50mM Tris pH8.0, 150 mM NaCl, 5 mM EDTA, 0.5% NP-40, 1 mM DTT, 1 mM PMSF, Complete EDTA-free from Roche Diagnostics, Mannheim, Germany) and samples were separated on 8–12% SDS gels, as has previously described [96,98,99]. Blotting onto activated PVDF membranes was achieved with Trans-Blot Turbo (Bio-Rad, Munich, Germany) and blocking and antibody incubations (1 h/RT or 16 h/4 °C depending on antibody) were performed in 5% milk powder or BSA in TBST or PBS. The detection of the luminescence signal of HRP-coupled secondary antibodies after the addition of Clarity Western ECL Substrate was performed using the ChemiDoc^TM^ imaging system (Bio-Rad). Equal loading of lysates was controlled by reprobing blots for housekeeping genes (Actin). At least n = 2 biological replicates were performed and representative results are shown. Results of the densitometric analyses of all Western blots can be found in the Supplementary Materials.

### 4.11. Statistical Analysis

Statistical analyses were performed using GraphPad Prism (version 9.3.1) as described [39]. Survival data were obtained from the USCS Xena server, visualized, and analyzed by GraphPad Prism (Log-rank/Mantel-Cox test; Hazard Ratio (Mantel-Haenszel)). For two groups, a paired or unpaired Student’s *t*-test, for more group analysis of variance (ANOVA) was performed. Unless stated otherwise, *p* values represent data obtained from two independent experiments conducted in triplicate. Statistical significance is represented in the figures as follows: * *p* < 0.05, ** *p* < 0.01, *** *p* < 0.001, **** *p* < 0.0001, and n.s. indicates not significant. A *p*-value that was less than 0.05 was considered statistically significant.

## Figures and Tables

**Table 1:** Comparison between the two studied groups, according to different parameters. Chi-square test U: Mann–Whitney test, t: Student *t*-test, *p*: *p*-value for comparing between the two groups *: Statistically significant at *p* ≤ 0.05.

	Cases (n = 40)	Control (n = 40)	Test of Sig.	*p*
Sex				
Male	8 (20%)	9 (22.5%)	χ^2^ = 0.075	0.785
Female	32 (80%)	31 (77.5%)		
Age (years)				
Mean ± SD.	60.9 ± 10.5	53.3 ± 8.2	t = 3.594 *	0.001 *
Median (Min–Max)	59 (42–83)	55 (35–72)		
VitD (ng/mL)				
All Cases				
Mean ± SD.	7.4 ± 4.5	28.7 ± 4.6	U = 0.0 *	<0.001 *
Median (Min–Max)	5.2 (3.3–18.1)	29.5 (20–40)		
Male				
Mean ± SD.	15.5 ± 1.7	23 ± 2	U = 0.0 *	<0.001 *
Median (Min–Max)	15.3 (13.6–18.1)	23 (20–25.5)		
Female				
Mean ± SD.	5.3 ± 1.7	30.3 ± 3.8	U = 0.0 *	<0.001 *
Median (Min–Max)	4.8 (3.3–9.5)	31 (23–40)		

**Table 2:** Overview of obtained results (shown in Figure 1) summarizing the histopathological differentiation, VitD serum levels, and VDR/Ki67 expression in the cancer patients and healthy controls. VDR expression in the cancer tissue was inversely correlated with the VitD serum levels. High VDR expression occurred in poorly differentiated, highly proliferative tumor tissues.

Differentiation	VitD ng/mL	VDR		Ki67 (IHC)
		IF	IHC	
Poor	4.1 ± 0.5	High	High	High
Moderate	7.3 ± 4.3	Med	Med	Med
Well	13.2 ± 3.4	Low	Low	Low
Control	29.12 ± 4.7	M = Low, F = High	M = Low, F = High	Low

## Data Availability

The cell line raw data required to reproduce these findings are available upon request. The clinical results shown here are based on data generated by the TCGA Research Network: https://www.cancer.gov/tcga, accessed on 1 October 2022.

## Supplementary Materials

The following supporting information can be downloaded at: https://www.mdpi.com/article/10.3390/ijms24054675/s1.
