# Supplementary material for: Exploiting Vitamin D Receptor and Its Ligands to Target Squamous Cell Carcinomas of the Head and Neck
Source: Int J Mol Sci. 2023 Feb 28;24(5):4675. doi: 10.3390/ijms24054675 (PMC10002563; doi:10.3390/ijms24054675)
Supplement: Supplementary file 1 [file ijms-24-04675-s001.zip › ijms-2188293-supplementary.pdf]

International Journal of *Molecular Sciences*

Exploiting Vitamin D Receptor and its Ligands to Target Squamous Cell Carcinomas of the Head and Neck

Laura Koll [1,†], Désirée Gül [1,†], Manal I. Elnouaem[2], Hanaa Raslan[2], Omneya R. Ramadan[2], Shirley K. Knauer[3], Sebastian Strieth[4], Jan Hagemann[1], Roland H. Stauber[1,*], and Aya Khamis[1,2,*]

1 Department of Otorhinolaryngology Head and Neck Surgery, Molecular and Cellular Oncology, University Medical Center, 55131 Mainz, Germany
2 Oral Pathology Department, Faculty of Dentistry, Alexandria University, 5372066 Alexandria, Egypt
3 Centre for Medical Biotechnology (ZMB/CENIDE), Institute for Molecular Biology, University Duisburg-Essen, Universitätsstraße, 45117 Essen, Germany
4 Department of Otorhinolaryngology, University Medical Center Bonn, 53127 Bonn, Germany
† These authors contributed equally to this work.
* Correspondence: ayakhamis@uni-mainz.de (A.K.); rstauber@uni-mainz.de (R.H.S.) Tel.: +49-6131176030 (A.K. & R.H.S)

Table S1. Antibodies used for Western Blot and immunofluorescence analyses.

Antigen	Host	Manufacturer and Article Number	Dilution (x-fold)
Western Blot Analysis			
**Actin**	rabbit	Sigma Aldrich, A2066	5000
**VDR**	mouse	Santa Cruz; sc-13133	1000
**pmTOR**	mouse	Cell Signaling; 5536	1000
**pAKT**	mouse	Cell Signaling; 3787	1000
**Rabbit (HRP coupled)**	goat	Cell Signaling; 7074	5000
**Mouse (HRP coupled)**	mouse	Cell Signaling; 7076	5000
Immunofluorescence			
VDR	rabbit	Abcam ab3508	200
**RXRα**	rabbit	Cell Signaling; 5388	200
Rabbit (FITC coupled)	goat	Dianova; 111-165-003	300
Rabbit (Cy3 coupled)	goat	Dianova; 111-165-062	300
Immunohistochemistry			
**VDR**	rabbit	Abcam ab3508	200
**Ki67**	mouse	Dako IR626	Ready to use
**DakoEnVision Flex (Linker)**	goat	Dako EnVision DM824	Ready to use

Table S2. Primer sequences

**Name**	**Sequence (5′ → 3′)**
**VDR_fw**	TACCGAGCTCGGATCCCCTGGGCTCCACTTACCTG
**VDR_rev**	CCTCGCCCTTGCTAGCGGAGATCTCATTGCCAAACAC
**ColPCR_pC3_fw**	CCCACTGCTTACTGGCTTAT
**ColPCR_pC3_rev**	AGCAGTACGATCTGGTCCT

Table S3. Clinical tumor patient data (n=40) showing gender, age, tumor differentiation, VDR expression in tumor tissue, Vitamin D level in the patient serum before or on day of surgery, and tumor site. TNM and Stage according to the UICC (8th edition).

No	Sex	Age	Diff	VDR exp.	VitD [ng/ml]	Site	T	N	M	Stage
1	Female	62	moderate	291	5,2	alveolar	T_2	N_0	M_0	II
2	Female	66	moderate	254	6,8	tongue	T_1	N_0	M_0	I
3	Female	55	moderate	228	9,43	tongue	T_1	N_0	M_0	I
4	Female	73	moderate	295	4,66	tongue	T_2	N_1	M_0	III
5	Female	42	poor	412	3,82	tongue	T_2	N_{2a}	M_0	IV
6	Female	82	moderate	90,8	5,48	tongue	T_2	N_0	M_0	II
7	Male	76	well	8,6	14,9	alveolar	T_1	N_0	M_0	I
8	Female	49	poor	486	4,22	tongue	T_3	N_1	M_0	III
9	Female	52	poor	4070	4,3	alveolar	T_4	N_0	M_0	IV
10	Female	72	moderate	3340	4,52	alveolar	T_2	N_0	M_0	II
11	Male	52	moderate	7,11	17,8	tongue	T_1	N_0	M_0	I
12	Female	65	well	2990	8,69	buccal mucosa	T_1	N_0	M_0	I
13	Female	48	moderate	250	6,39	buccal mucosa	T_2	N_0	M_0	II
14	Female	53	moderate	659	4,11	tongue	T_2	N_0	M_0	II
15	Male	58	moderate	8,37	13,6	buccal mucosa	T_1	N_0	M_0	I
16	Female	55	moderate	294	4,66	tongue	T_2	N_1	M_0	III
17	Female	77	poor	409	3,82	Lip	T_4	N_{2a}	M_0	IV
18	Female	62	moderate	90,8	5,48	tongue	T_3	N_0	M_0	III
19	Male	58	well	8,3	14,9	buccal mucosa	T_1	N_0	M_0	I
20	Female	60	poor	493	4,22	Lip	T_2	N_1	M_0	III
21	Female	65	moderate	429	6,29	alveolar	T_3	N_0	M_0	III
22	Female	58	moderate	353	8,45	tongue	T_2	N_0	M_0	II
23	Female	60	moderate	134	7,25	tongue	T_1	N_0	M_0	I
24	Female	61	moderate	251	5,18	tongue	T_1	N_1	M_0	III
25	Female	53	poor	102	8,24	tongue	T_2	N_{2b}	M_0	IV
26	Female	52	moderate	354	9,28	tongue	T_1	N_0	M_0	I
27	Male	53	well	151	7,87	alveolar	T_2	N_0	M_0	II
28	Female	58	poor	187	5,74	tongue	T_2	N_1	M_0	III
29	Female	65	poor	319	9	alveolar	T_3	N_0	M_0	III
30	Female	57	moderate	488	7,34	alveolar	T_1	N_0	M_0	I

31	Male	65	moderate	388	7,78	tongue	T_1	N_0	M_0	I
32	Female	64	well	452	9,2	buccal mucosa	T_2	N_0	M_0	II
33	Female	54	moderate	409	5,58	buccal mucosa	T_2	N_0	M_0	II
34	Female	63	moderate	406	9,27	tongue	T_2	N_0	M_0	II
35	Male	68	moderate	307	4,43	buccal mucosa	T_1	N_0	M_0	I
36	Female	63	moderate	462	9	tongue	T_1	N_0	M_0	I
37	Female	70	poor	150	6,29	Lip	T_4	N_1	M_0	IV
38	Female	55	moderate	151	8,25	tongue	T_2	N_0	M_0	II
39	Male	58	well	347	4,27	buccal mucosa	T_1	N_0	M_0	I
40	Female	60	poor	359	9,38	Lip	T_2	N_0	M_0	II

Table S4. Control cases data (n=40) showing gender, age, and Vitamin D level in the control group.

No	Sex	Age	VitD [ng/ml]
1	Female	39	25
2	Female	57	31,5
3	Female	62	30,5
4	Female	60	26
5	Female	58	32
6	Female	35	34
7	Male	50	35
8	Female	72	40
9	Female	65	25
10	Female	55	23
11	Male	45	25
12	Female	58	31,1
13	Female	65	30,5
14	Female	59	32,7
15	Male	45	34,5
16	Female	61	35
17	Female	40	21
18	Female	45	22
19	Male	35	23
20	Female	50	25,5
21	Female	55	25
22	Female	52	33
23	Female	53	25
24	Female	56	25
25	Female	46	31
26	Female	55	33
27	Male	50	25,5
28	Female	45	32

29	Female	54	28,25
30	Female	54	31
31	Male	48	25
32	Female	56	32
33	Female	48	30
34	Female	56	30
35	Male	60	25
36	Female	59	32,28
37	Female	60	29
38	Female	48	28,5
39	Male	60	29
40	Female	60	28

Table S5. Classification of the studied cases according to different parameters (n=40). Qualitative data were described using numbers and percentages, while quantitative data were expressed in mean ± SD and median (Min. - Max.)

**Differentiation**	**No. (%)**
Well	6 (15%)
Moderate	24 (60%)
Poor	10 (25%)
**Receptor expression**	Value
**Tumor**	
Mean ± SD.	717.4 ± 1181.5
Median (Min. – Max.)	275.5 (7 – 4075)
**Control**	
Mean ± SD.	1990.3 ± 2910.1
Median (Min. – Max.)	796.5 (99 – 10100)
**Site**	**No. (%)**
Alveolar	8 (20%)
Tongue	8 (20%)
Buccal mucosa	4 (10%)
Lip	20 (50%)

Table S6. RNA sequencing analysis of selected nuclear receptors. All values in FPKM.

Pica	Wt			Res		
Estrogen receptor-α	0,086986	0,156135	0,0937655	0,12447	0,300349	0,0756134
Estrogen receptor- β	0,456702	0,355594	0,485498	0,590792	0,743111	0,206664

Estrogen-related receptor- α	78,5708	49,7873	51,6182	39,9401	37,9974	49,9846
Hepatocyte nuclear factor-4-γ	0,707849	0,588592	0,75755	0,130655	0,390305	0,0678893
Rev-ErbA α	8,59431	5,12433	4,47826	4,75481	4,55256	5,74921
Rev-ErbA β	8,07561	7,54943	6,36084	7,84262	7,67867	6,58514
Liver X receptor- β	47,9511	34,3668	31,5217	32,1372	35,2124	40,8427
Liver X receptor- α	2,544	3,26562	2,79666	2,8456	3,30791	4,11189
Testicular receptor 2	6,73481	5,85692	7,81062	11,3826	15,6774	11,1582
Testicular receptor 4	5,51314	4,90238	4,59446	6,63569	7,60539	5,72504
Glucocorticoid receptor	13,5492	16,2514	12,3936	18,472	17,0918	20,8316
Nerve Growth factor I β	4,12304	4,27002	4,8996	4,50531	8,12497	8,35808
Nuclear Receptor Subfamily 4 Group A Member 2	0,377373	0,200647	0,122973	0,954419	1,14045	1,17038
Neuron-derived orphan receptor 1	0,101247	0,17567	0,195954	0,198881	0,349486	0,243172
Liver receptor homolog-1	0,232363	0,354592	0,447433	0,598797	0,640391	0,375258
Germ cell nuclear factor	1,45105	1,18439	1,23121	1,75102	1,66925	1,07981
Peroxisome proliferator-activated receptor- α	4,73668	4,00861	3,18864	2,33052	3,19441	2,94178
Peroxisome proliferator-activated receptor- β /δ	11,1699	6,65914	7,88922	7,33305	6,59849	8,66761
Peroxisome proliferator-activated receptor- γ	6,18746	7,44107	5,52167	7,46862	6,75023	8,36472
Retinoic acid receptor- α	13,8344	7,73934	8,64073	6,90167	7,92416	9,51698
Retinoic acid receptor- γ	44,4628	42,6432	41,904	30,3559	29,1827	39,9099
RAR-related orphan receptor- α	0,147742	0,246185	0,148643	0,121437	0,114879	0,056787
Retinoid X receptor- α	96,4527	63,4225	65,2665	39,9713	43,2624	54,3649
Retinoid X receptor- β	24,9242	21,9572	23,9392	17,0627	17,579	15,4295
Thyroid hormone receptor- α	10,283	6,00473	6,64359	2,90511	2,47679	3,97976
Thyroid hormone receptor-β	0,922238	0,530259	0,481687	0,30015	0,388194	0,108029
Vitamin D receptor	20,9758	19,8167	16,3955	10,8225	10,2558	15,1325

Supplementary Figures

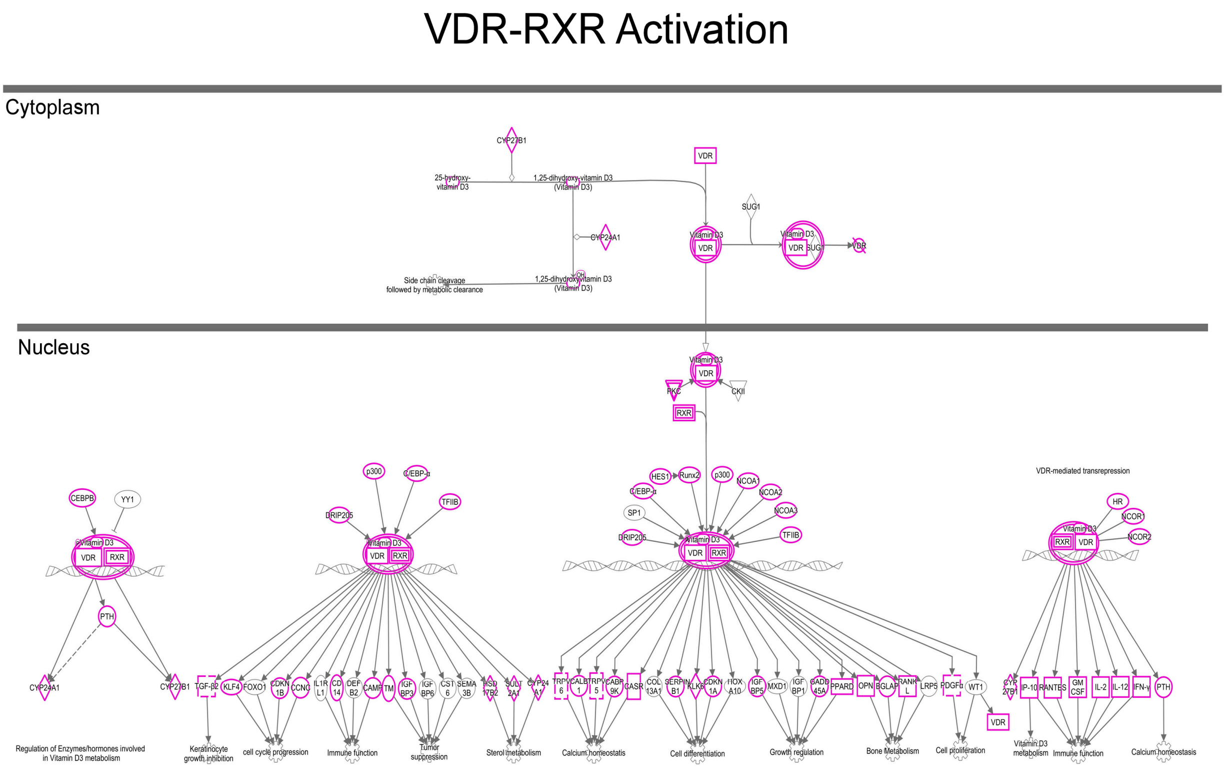

Figure S1. Molecular network of VDR-RXR activation interaction partners. Ingenuity pathway analysis of known direct VDR-RXR-interaction partners which are involved in the interaction mechanism. Cellular localization indicated.

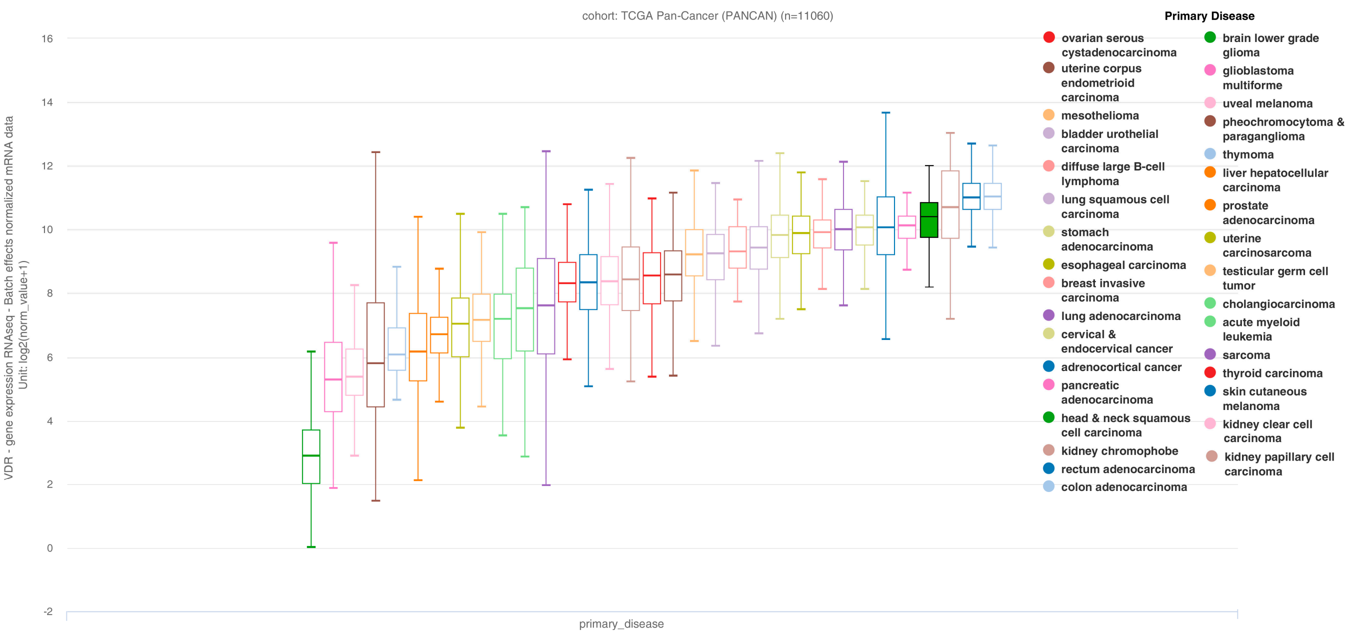

Figure S2. VDR mRNA expression in different tumor types according to the PANCAN data set obtained from *The Cancer Genome Atlas* (TCGA). VDR is highly expressed not only in colon adenocarcinomas, but also in HNSCC (green) supporting a pathobiological relevance of VDR for this tumor entity.

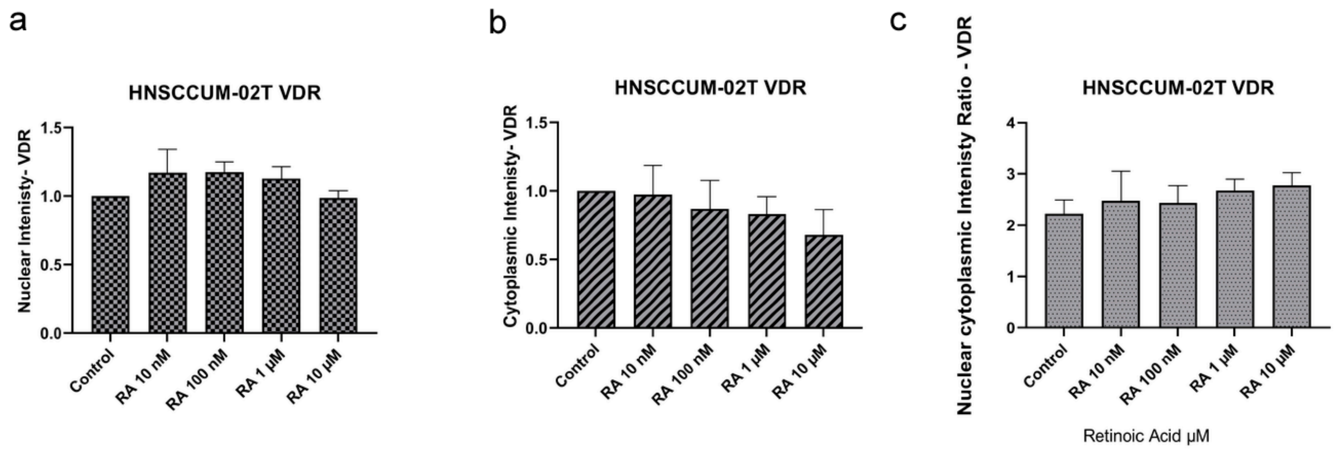

Figure S3. VDR translocation after retinoic acid (RA) treatment was automatically quantified by high-throughput microscopy and normalized to untreated controls. Statistical analysis of the (a) nuclear intensity (b) cytoplasmic intensity (c) nucleus-to-cytoplasm ratio (N/C) reveals non-significantly changes in the presence of retinoic acid compared to untreated controls indicating a lack of cytoplasm-to-nuclear translocation.

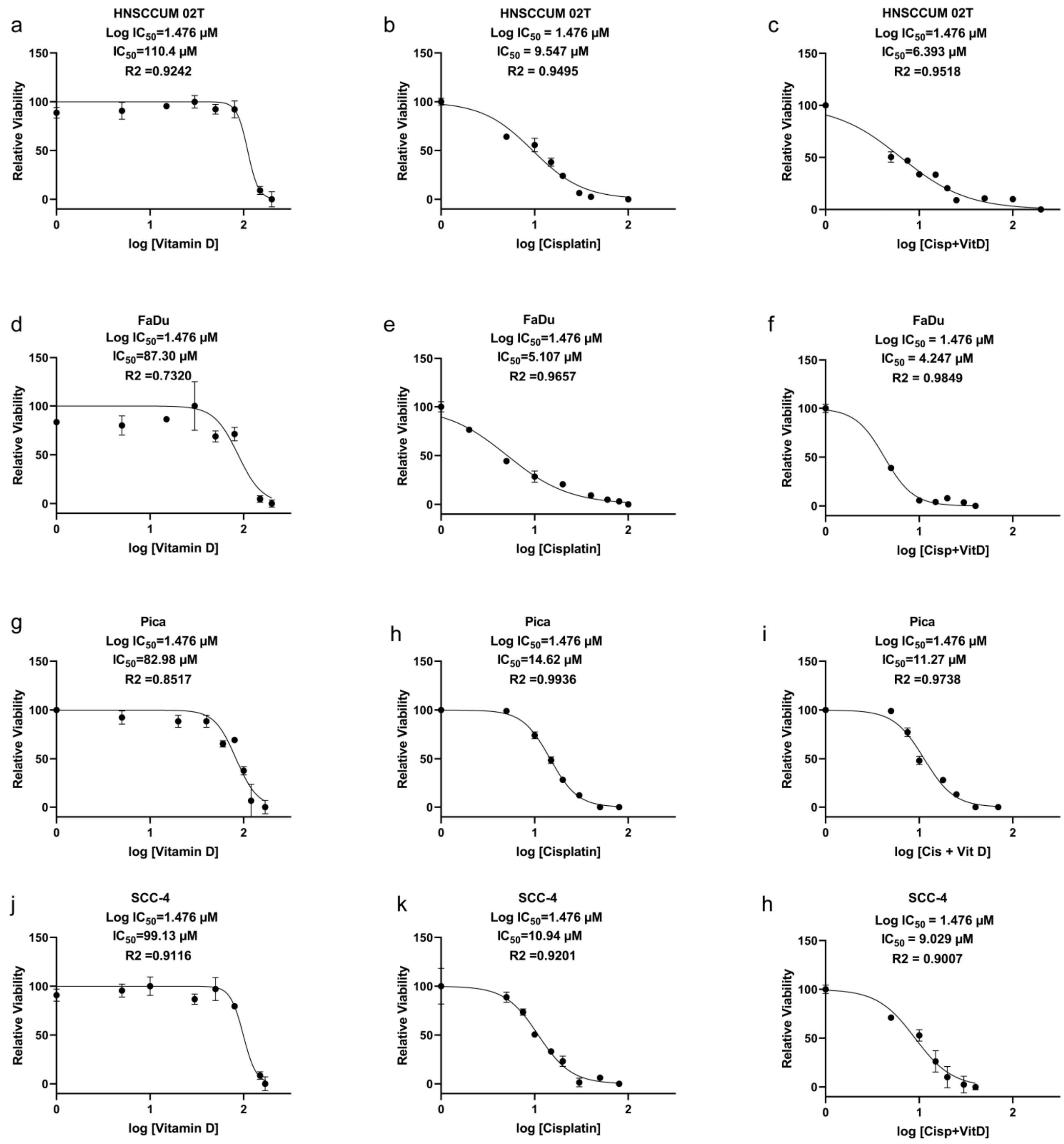

Figure S4. Dose-response curves and IC50 values for HNSCCUM-02T (a-c), FaDu (d-f), Pica cells (g-i) , and SCC-4 (j-h).

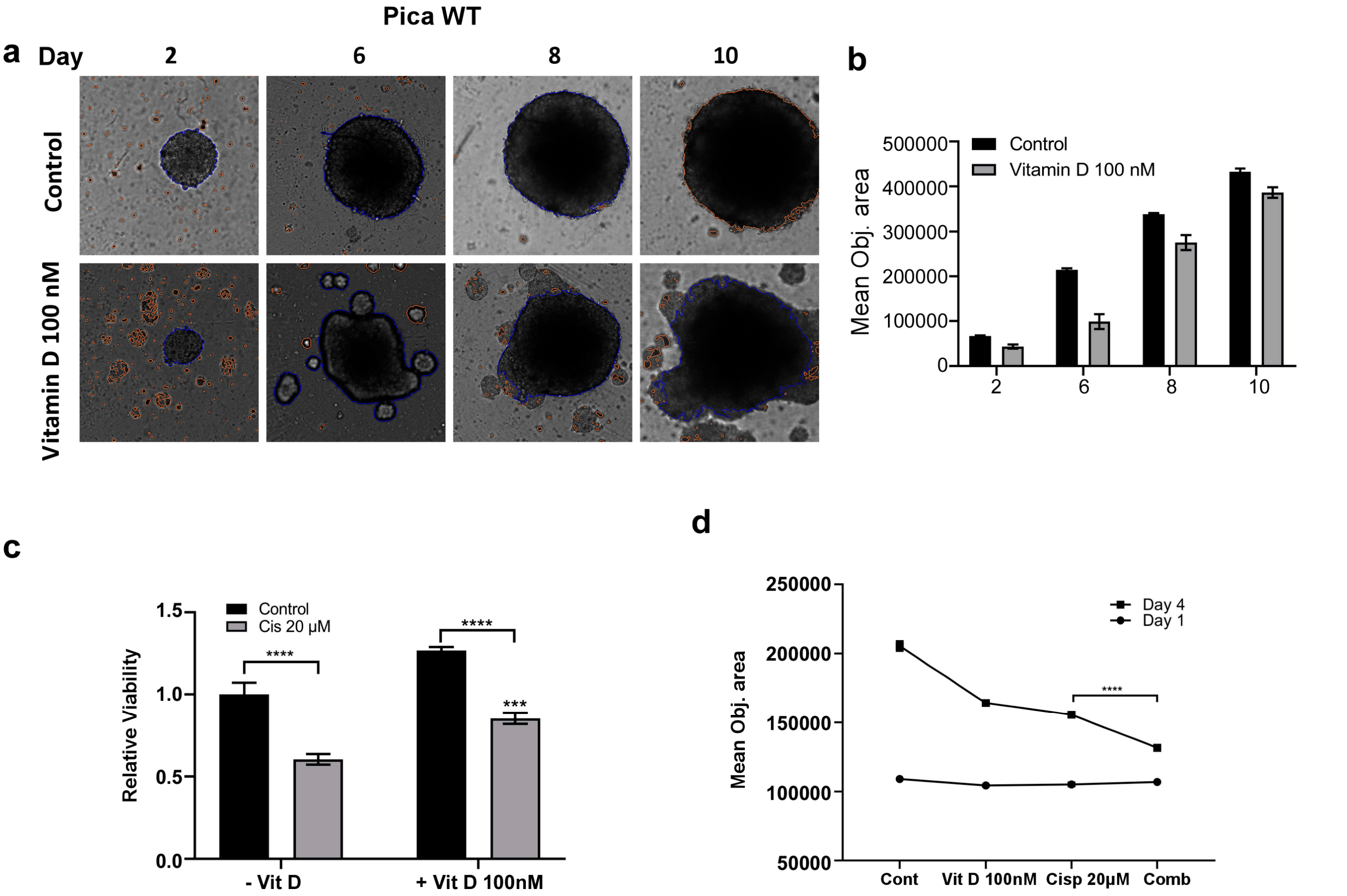

Figure S5. Assessing the impact of VitD/VDR targeting in HNSCC 3D tumor spheroids. (a) & (b) Automated high-content microscopy was used to visualize (a) and quantify (b) tumor spheroid growth of Pica$_{wt}$ after VitD treatment. Spheroid formation was observed over 10 days without (control) and with the addition of 100nM VitD. The mean object area of Pica$_{wt}$ spheroids (n=8) was automatically determined by HCS Array Scan VTI. (c) VitD/cisplatin treatment synergistically inhibits cell proliferation in Pica$_{wt}$ cells. Spheroids were treated with VitD (100nM), cisplatin (20µM), and a combination of both for 72 hrs, and viability was normalized to untreated controls. (d) Synergistically killing effect of VitD/cisplatin is also reflected by decreasing spheroid size (area). Mean object sizes of Pica spheroids (n=8) were automatically determined by HCS microscope Array Scan VTI after VitD, cisplatin, and combinational treatment (72 hrs) and normalized to untreated control.

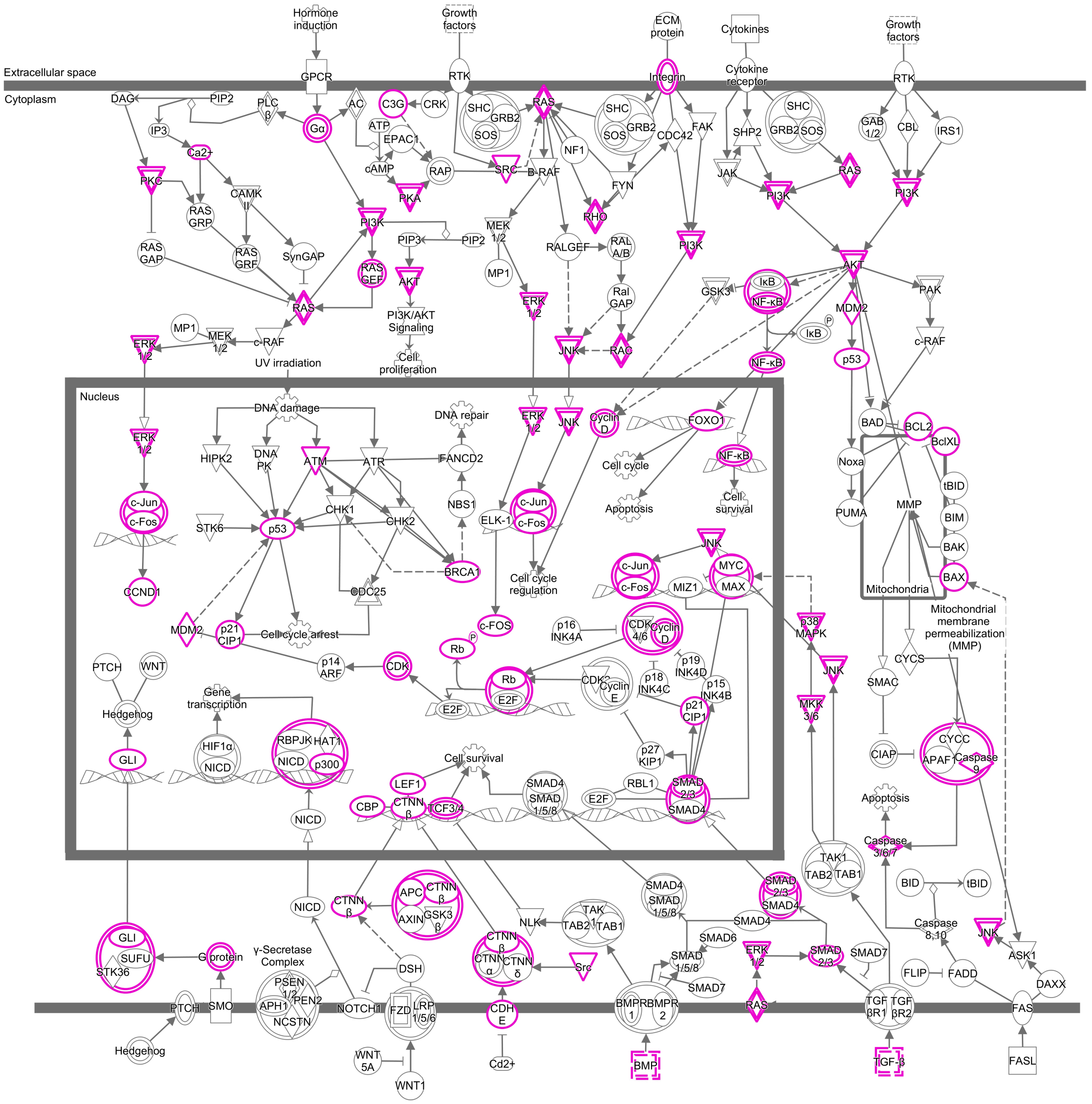

Figure S6. Ingenuity Pathway Analysis Molecular mechanism interaction partners involved in cancer diseases. Proteins potentially affected by VitD/VDR activation are highlighted in pink. Cellular localization indicated.

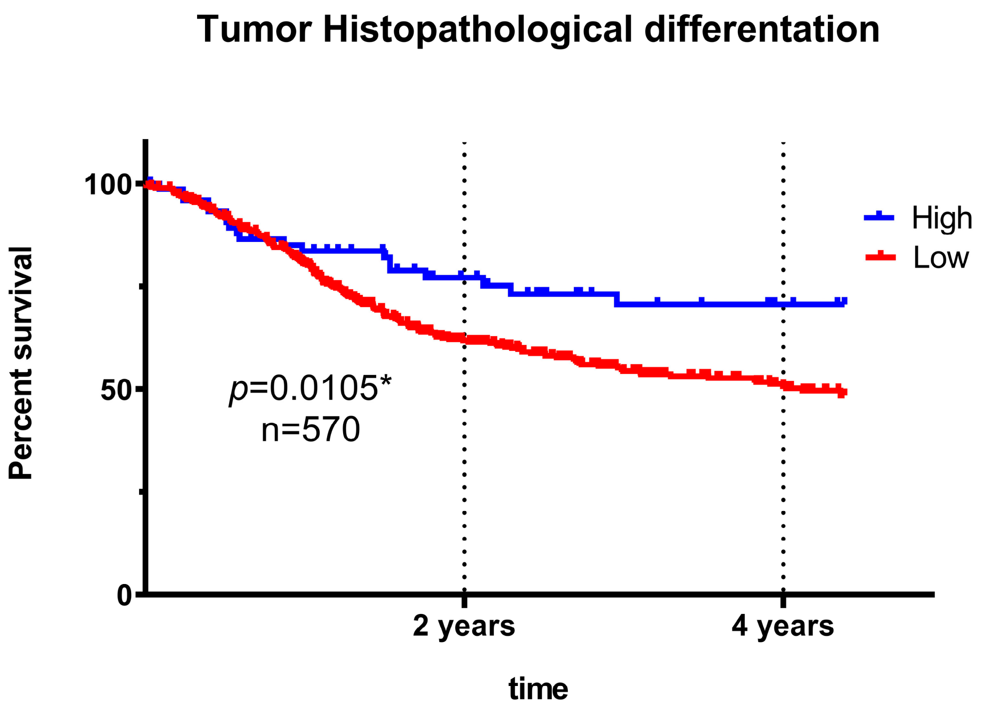

Figure S7. Overall survival of HNSCC patients is correlating with tumor histopathological differentiation. Data were obtained from TCGA dataset (n=570). High differentiation (blue) includes G1, and low (red) includes G2&G3.
